# Supplementary material for: Mitochondrial control of amino acid catabolism by a fasting-inducible mitochondrial carrier
Source: Sci Adv. 2026 Jul 31;12(31):eaef0140. doi: 10.1126/sciadv.aef0140 (PMC13426421; doi:10.1126/sciadv.aef0140)
Supplement: Supplementary file 1 — Figs. S1 to S7 Tables S1 to S10 [file sciadv.aef0140_sm.pdf]

Science Advances
AAAS

Supplementary Materials for

Mitochondrial control of amino acid catabolism by a fasting-inducible mitochondrial carrier

Satoshi Oikawa *et al.*

Corresponding author: Shingo Kajimura, skajimur@bidmc.harvard.edu

Sci. Adv. **12**, eaef0140 (2026)
DOI: 10.1126/sciadv.aef0140

This PDF file includes:

Figs. S1 to S7
Tables S1 to S10

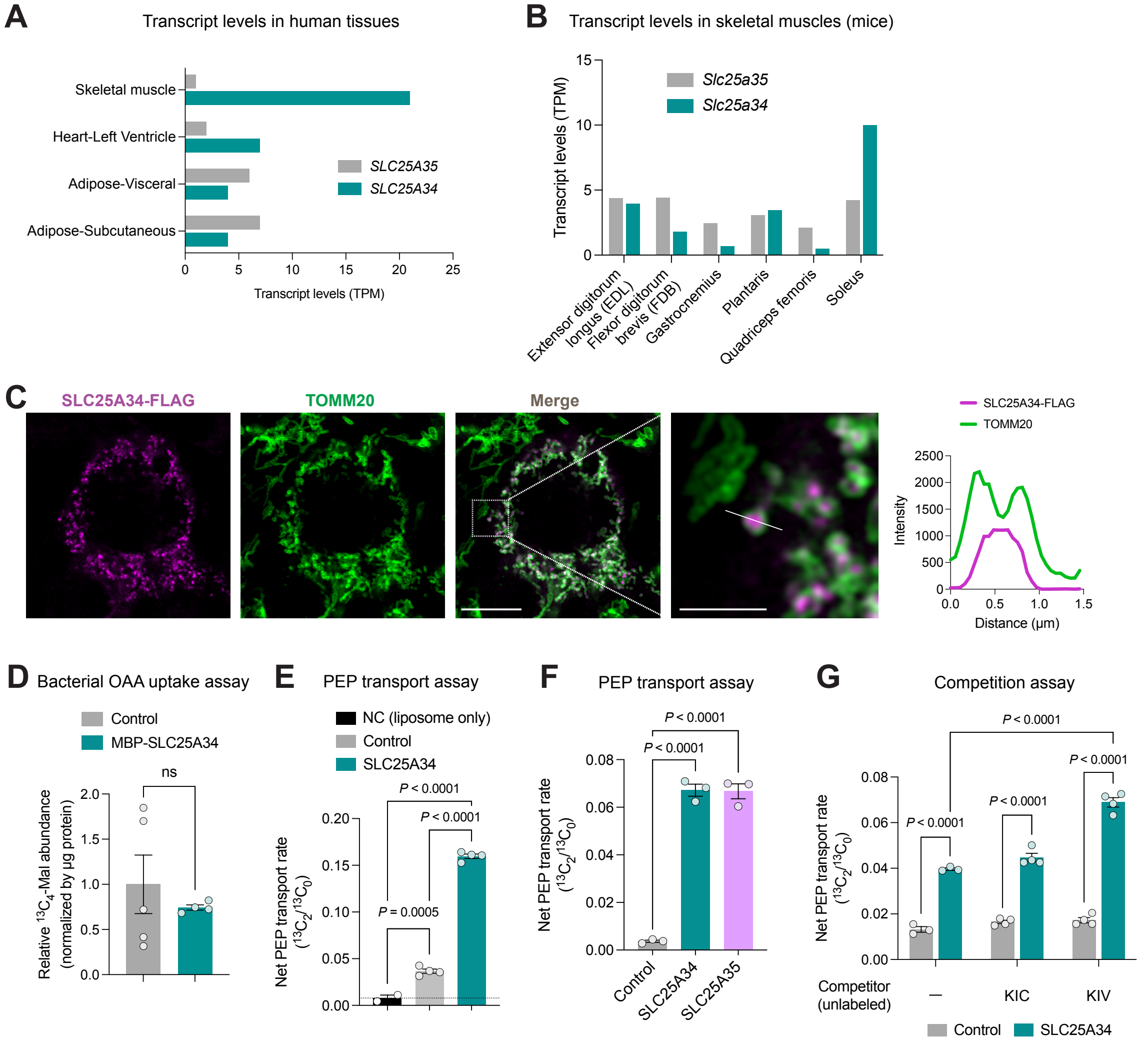

Figure S1. Molecular characterization of SLC25A34, related to Figure 1. (A) Transcript levels (TPM) of *SLC25A34* and *SLC25A35* in the indicated human tissues. Data were obtained from GTEx Portal (https://www.gtexportal.org/home/). **(B)** Transcript levels (TPM) of *Slc25a34* and *Slc25a35* in the extensor digitorum longus (EDL), flexor digitorum brevis (FDB), gastrocnemius, plantaris, quadriceps femoris, soleus (GEO: GSE100505). **(C)** Representative immunofluorescence image of HEK 293T cells with SLC25A34-FLAG (magenta) and TOMM20 (mitochondrial outer membrane; green). Scale bars, 20 μm. The inset shows higher-magnification views of the boxed regions. Scale bar, 5 μm. The graph panel shows the fluorescence intensity along the solid white line. **(D)** Bacterial oxaloacetate (OAA) uptake assay. Bacteria expressing the endogenous MBP protein alone (control) or MBP-SLC25A34 were incubated with $^{13}C_4$-OAA (1 mM) for 30 min at 37°C. Derivatized $^{13}C_4$-malate, a downstream metabolite of OAA, was analyzed by LC-MS and normalized to the protein levels in each sample. n = 5 for control and n = 4 for MBP-SLC25A34. **(E)** PEP transport assay in proteo-liposomes reconstituted with purified SLC25A34 or control-eluates. Background signals obtained from protein-free empty control liposomes (*i.e.,* liposome lipids only) were subtracted, as these signals represent non-specific association with the liposomes. n = 2 for NC (liposome lipids only), n = 4 for control and SLC25A34. **(F)** PEP transport assay in proteo-liposomes reconstituted with purified SLC25A34 or SLC25A35 or control-eluates. n = 3 per group. **(G)** Competition assay. An excess amount of indicated metabolites (50 mM) was added to the assay. n = 3 for non-competitor and n = 4 for KIC and KIV. Bars represent mean ± s.e.m. *P* values were calculated by unpaired *t*-test (D), one-way ANOVA with Tukey's multiple comparisons test (E and F) and two-way ANOVA with Tukey's multiple comparisons test (G).

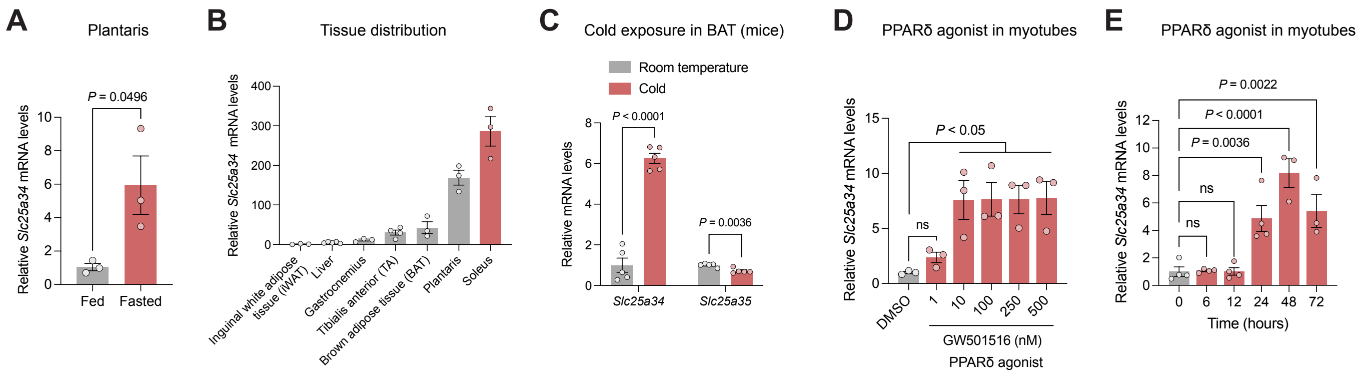

Figure S2. Regulation of SLC25A34 expression, related to Figure 2. (A) Relative mRNA levels of *Slc25a34* in plantaris muscles from wild-type mice fasted for 24 hours, measured by qPCR. n = 3 per group. **(B)** Relative mRNA levels of *Slc25a34* in the indicated tissues from wild-type mice, as measured by qPCR. n = 3 for inguinal white adipose tissue (iWAT), gastrocnemius, brown adipose tissue (BAT), plantaris, soleus; n = 4 for tibialis anterior (TA); n = 5 for liver. **(C)** Relative mRNA levels of *Slc25a34* and *Slc25a35* in brown adipose tissue (BAT) from mice that were kept at room temperature (RT) or exposed to cold (4°C) for 3 days (GEO: GSE70437). n = 5 per group. **(D)** Dose-dependent changes in *Slc25a34* expression levels upon PPARδ agonist treatment. Differentiated C2C12 myotubes were treated with the indicated concentrations of GW501516 for 24 hours. Relative *Slc25a34* mRNA levels were measured using qPCR. n = 3 per group. **(E)** Time-dependent changes in *Slc25a34* expression levels following PPARδ agonist treatment. Differentiated C2C12 myotubes were treated with GW501516 (100 nM) for the indicated times (0-72 hours). Relative *Slc25a34* mRNA levels were measured using qPCR. n = 4 for 0, 6, 12, 24 hours and n = 3 for 48 and 72 hours. Bars represent mean ± s.e.m. *P* values were calculated by unpaired *t*-test (A and C) and one-way ANOVA with Dunnett's multiple comparisons test (D and E).

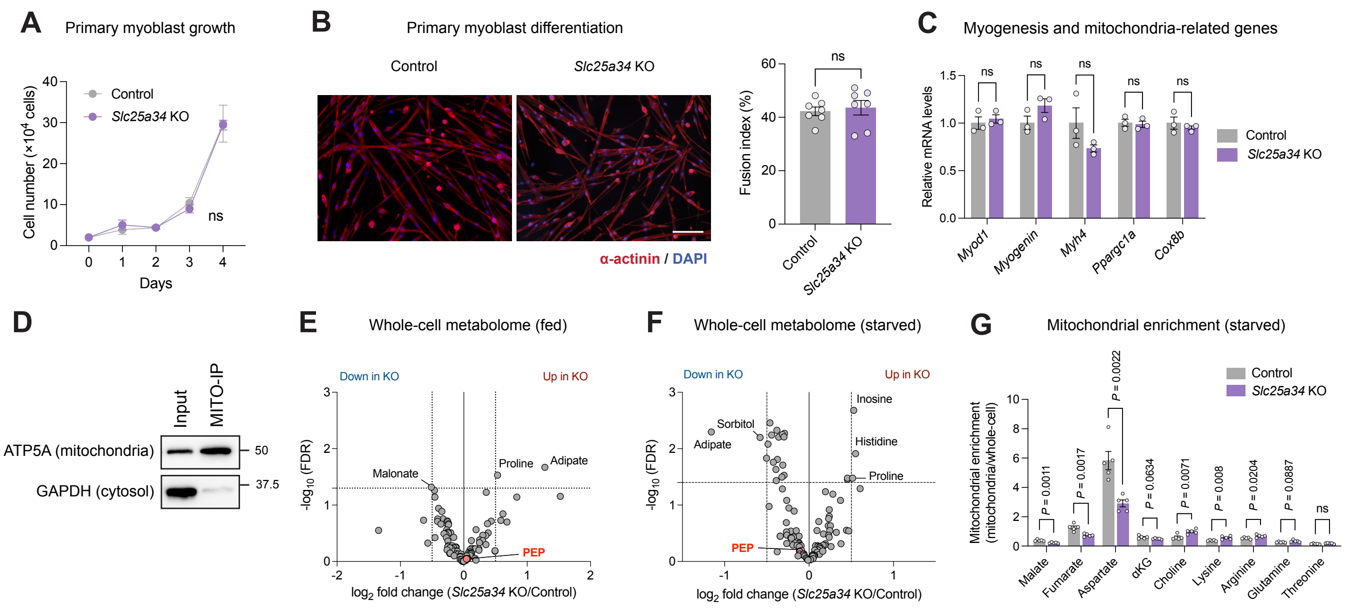

Figure S3. Myogenesis and metabolomics in *Slc25a34* KO primary myotubes, related to Figure 3. (A) Growth of control and *Slc25a34* KO primary myoblasts. n = 4 at all time points. **(B)** Representative immunofluorescence images of control and *Slc25a34* KO primary myotubes stained for α-actinin and DAPI. The fusion index was manually determined by calculating the ratio of nuclei in α-actinin-positive myotubes containing more than two nuclei to the total number of nuclei. n = 7 per group. **(C)** Relative expression levels of myogenesis- and mitochondria-related marker genes in control and *Slc25a34* KO primary myotubes. n = 3 per group. **(D)** Mitochondria purification using the MITO-Tag system. Mitochondria were isolated from primary myotubes expressing MITO-Tag (3xHA-EGFP-OMP25) using magnetic beads and subjected to immunoblotting to detect ATP5A (a mitochondrial marker) and GAPDH (a cytosolic marker). **(E)** Whole-cell metabolomics of control or *Slc25a34* KO primary myotubes under a nutrient-replete culture condition (25 mM glucose, 5% horse serum, and amino acids). n = 5 per group. **(F)** Whole-cell metabolomics of control or *Slc25a34* KO primary myotubes under a nutrient-deprived culture condition (HBSS containing 5.5 mM glucose). n = 5 per group. **(G)** Mitochondrial metabolite enrichment in control or *Slc25a34* KO primary myotubes cultured in a nutrient-deprived condition. Mitochondrial enrichment was calculated as the ratio of mitochondrial to whole-cell intensity of the indicated metabolites. n = 5 per group. Bars represent mean ± s.e.m. *P* values were calculated by two-way ANOVA with Tukey's multiple comparisons test (A), unpaired *t*-test (B, C, G) and unpaired *t*-test with Benjamini-Hochberg FDR correction (E and F).

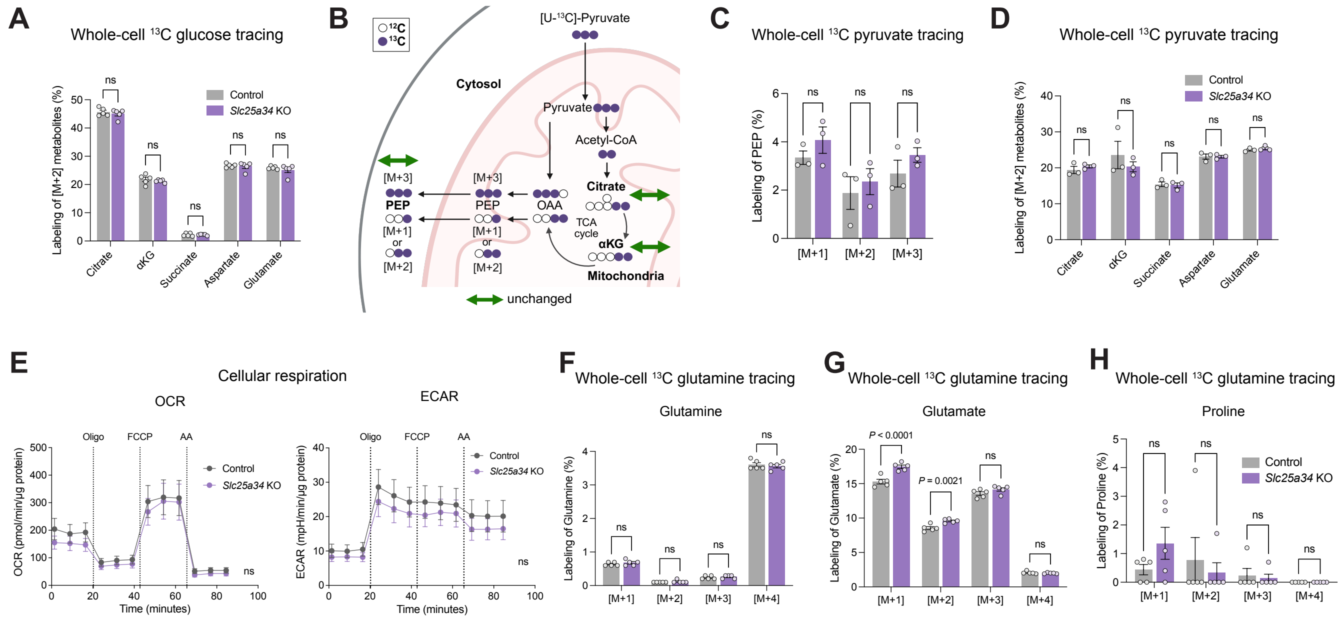

Figure S4. Stable isotope studies in *Slc25a34* KO primary myotubes, related to Figure 3. (A) Whole-cell [U-^{13}C]-glucose tracing in control or *Slc25a34* KO primary myotubes under a nutrient-deprived culture condition (HBSS). The labeling of the indicated ^{13}C-labeled [M+2] metabolites (%) is shown on the Y-axis. n = 5 per group. **(B)** Schematic of [U-^{13}C]-pyruvate tracing. Control or *Slc25a34* KO primary myotubes were cultured in a nutrient-deprived medium with HBSS containing [U-^{13}C]-pyruvate (1 mM) (green arrow, no change). Created in BioRender. Oikawa, S. (2026) https://BioRender.com/r8ll82a. **(C)** Whole-cell [U-^{13}C]-pyruvate tracing in control or *Slc25a34* KO primary myotubes cultured in a nutrient-deprived medium. The labeling of the indicated ^{13}C-labeled PEP (%) is shown on the Y-axis. n = 3 per group. **(D)** Whole-cell [U-^{13}C]-pyruvate tracing in control or *Slc25a34* KO primary myotubes in a nutrient-deprived medium, HBSS containing 1 mM [U-^{13}C]-pyruvate. The labeling of the indicated ^{13}C-labeled [M+2] metabolites (%) is shown on the Y-axis. n = 3 per group. **(E)** Cellular respiration in control and *Slc25a34* KO primary myotubes. Cellular oxygen consumption rate (OCR) and extracellular acidification rate (ECAR) were measured using the Seahorse XFe Extracellular Flux Analyzer. n = 9 per group. **(F)** [U-^{13}C]-glutamine tracing in control or *Slc25a34* KO cells under a nutrient-deprived medium condition. The labeling of the indicated ^{13}C-labeled glutamine (%) is shown. n = 5 per group. **(G)** [U-^{13}C]-glutamine tracing in (F). The labeling of the indicated ^{13}C-labeled glutamate (%) is shown. n = 5 per group. **(H)** [U-^{13}C]-glutamine tracing in (F). The labeling of the indicated ^{13}C-labeled proline (%) is shown. n = 5 per group. Bars represent mean ± s.e.m. *P* values were calculated by unpaired *t*-test (A and D), two-way ANOVA with Tukey's multiple comparisons test (C, F, G and H) and two-way repeated-measures ANOVA with Tukey's multiple comparisons test (E).

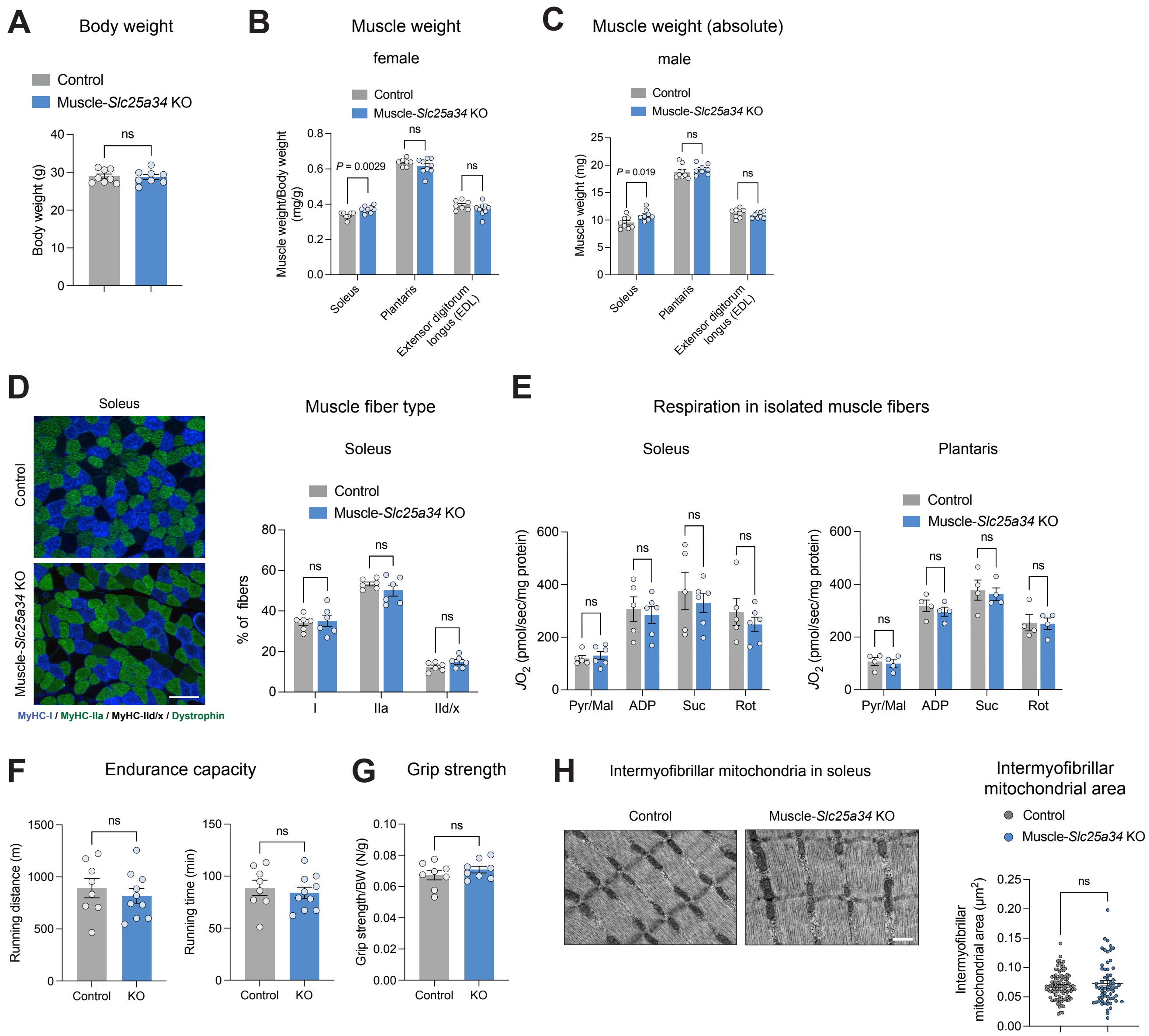

Figure S5. Characterization of muscle-*Slc25a34* KO mice, related to Figure 4. (A) Body weight in male control and muscle-*Slc25a34* KO mice fed a regular diet at 16 weeks old. n = 8 per group. **(B)** Muscle weight of the soleus, plantaris, and EDL in female control and muscle-*Slc25a34* KO mice at 17 weeks old. Muscle weight (mg) was normalized to the body weight (g). n = 7 for control and n = 9 for KO mice. **(C)** Absolute muscle weight of soleus, plantaris, and EDL in male control and muscle-*Slc25a34* KO mice at 16 weeks old. n = 8 per group. **(D)** Muscle fiber typing in soleus from male control and muscle-*Slc25a34* KO mice. n = 6 per group. **(E)** Respiratory function in isolated muscle fibers from soleus (left) and plantaris (right) of male control and KO mice. n = 5 for control and n = 6 for KO in soleus, n = 4 per group for plantaris. **(F)** Endurance performance in control and muscle-*Slc25a34* KO mice. Endurance exercise capacity assessed by running distance (left) and time (right) in male control and muscle-*Slc25a34* KO mice. n = 8 for control and n = 10 for KO. **(G)** Four-limb grip strength in male control and muscle-*Slc25a34* KO mice. n = 8 per group. Grip strength (N) was normalized to the body weight (g). **(H)** Representative electron microscopy images of intermyofibrillar mitochondrial structures in the soleus of male control and muscle-*Slc25a34* KO mice at 18 weeks old. Right: Quantification of intermyofibrillar mitochondrial area. Scale bar, 2 μm. n = 92 for control and 63 for KO collected from n = 2 mice per group. Bars represent mean ± s.e.m. *P* values were calculated by unpaired *t*-test (A, B, C, D, F, G and H) and two-way repeated-measures ANOVA with Tukey's multiple comparisons test (E).

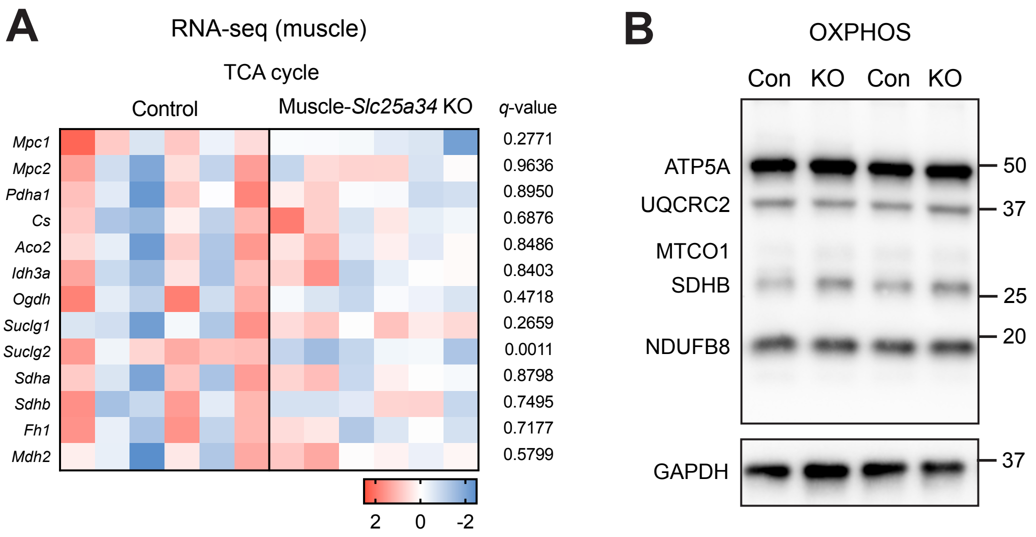

Figure S6. Molecular characterization of muscle-*Slc25a34* KO mice, related to Figure 5. **(A)** Relative mRNA levels of TCA cycle-related genes in the soleus muscles of male control and muscle-*Slc25a34* KO mice at 18 weeks old. Data represented as *Z*-score heatmaps for each gene in each sample, based on RNA-seq analysis. n = 6 per group. Red indicates elevated expression and blue indicates decreased expression. **(B)** Oxidative phosphorylation (OXPHOS) protein levels in the soleus muscle of male control and muscle-*Slc25a34* KO mice. Immunoblotting was performed to detect OXPHOS components (ATP5A, UQCRC2, MTCO1, SDHB and NDUFB8) and GAPDH. *q*-values were calculated by Wald test with Benjamini-Hochberg FDR correction (A).

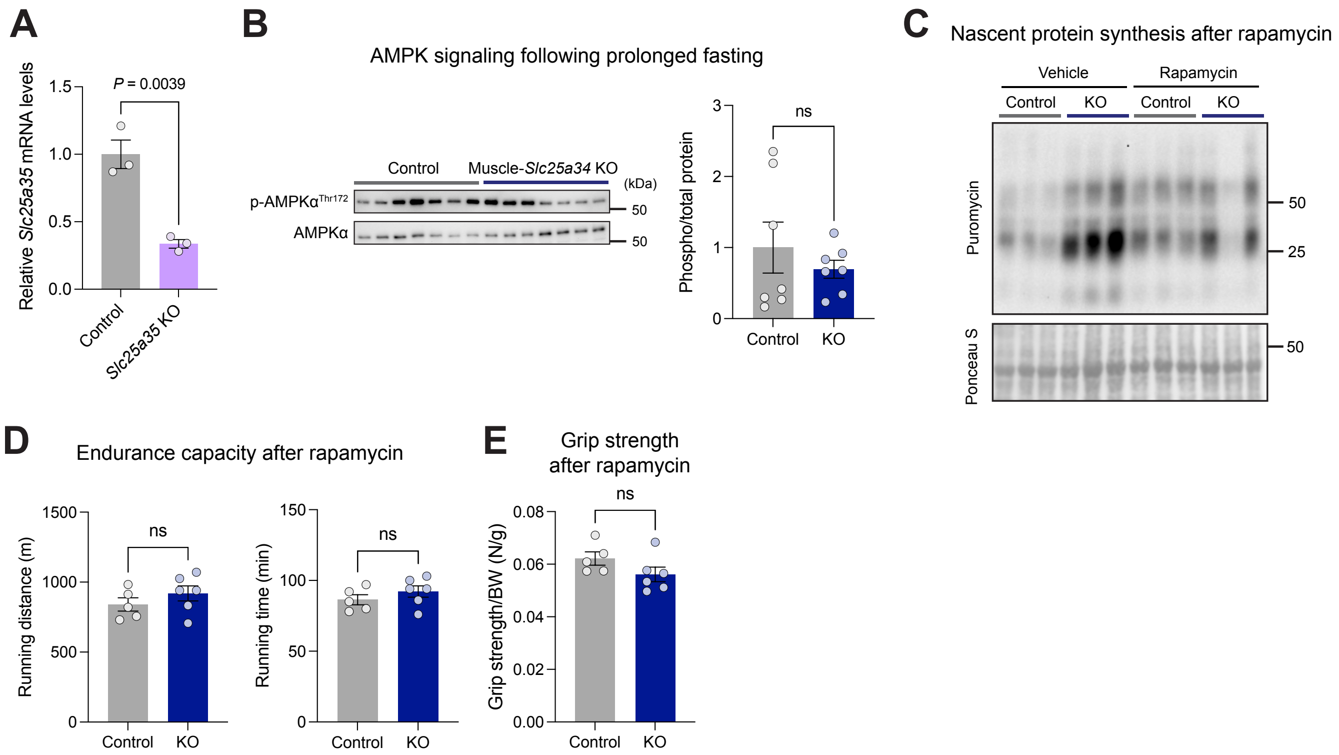

Figure S7. Molecular and physiological analyses of muscle-*Slc25a34* KO mice, related to Figure 6. **(A)** Relative mRNA levels of *Slc25a35* in control and *Slc25a35* KO primary myotubes. *Slc25a35*$^{flox/flox}$ cells were differentiated for 2 days, infected with adenovirus-GFP (Control) or adenovirus-Cre (KO), and analyzed by qPCR 2 days later. n = 3 per group. **(B)** AMPK signaling in the soleus muscle after 24 hours of fasting. Immunoblotting was performed to detect phosphorylated-AMPK (Thr172). Phosphorylated protein levels were normalized to the corresponding total protein levels. n = 7 per group. **(C)** Representative immunoblot of puromycin-labeled proteins in the soleus muscles of male control and muscle-*Slc25a34* KO mice following rapamycin treatment, assessed by the SUnSET assay. The total protein content (Ponceau S staining) was used as a loading control. **(D)** Endurance performance in control and muscle-*Slc25a34* KO mice following rapamycin treatment. Endurance exercise capacity assessed by running distance (left) and running time (right) in male control and muscle-*Slc25a34* KO mice. n = 5 for control and n = 6 for muscle-*Slc25a34* KO. **(E)** Four-limb grip strength in male control and muscle-*Slc25a34* KO mice following rapamycin treatment. n = 5 for control and n = 6 for muscle-*Slc25a34* KO. Grip strength (N) was normalized to the body weight (g). Bars represent mean ± s.e.m. *P* values were calculated by unpaired *t*-test (A, B, D and E).

Table S1

Differential mitochondrial metabolites in control and *Slc25a34* KO primary myotubes under a fed condition (*P*-value < 0.05), related to Figure 3.

Metabolite	FC	Log_2(FC)	*P*-value	$-log_{10}$(FDR)
Guanosine	2.1291	1.090217	0.004156	0.351972
Adipate	0.6224	-0.68407	0.004525	0.616058
N-Acetylaspartate	1.4307	0.51675	0.004686	0.776947
Citrate	0.6995	-0.51565	0.005481	0.833824
Choline	1.6641	0.73476	0.013059	0.553672
Cytidine	1.5089	0.593514	0.016549	0.529991
Uridine	2.0105	1.007585	0.019204	0.532312
Oxalate	0.7853	-0.34864	0.022153	0.52828
Inosine	1.6680	0.738149	0.02789	0.479405
Glutathione reduced	2.1308	1.091385	0.028267	0.519336
Malonate	0.8566	-0.22334	0.034897	0.469225
Carnosine	1.5247	0.608488	0.036524	0.48722
Fad	1.3527	0.43581	0.037001	0.516344
N-Acetylmethionine	1.4299	0.515932	0.038264	0.53395
N-Acetylglutamate	1.3246	0.40558	0.041589	0.527732
S-Adenosylhomocysteine	2.2248	1.153702	0.04203	0.551177
Acetoacetate	0.8150	-0.29508	0.046787	0.530941

Table S2

Differential whole-cell metabolites in control and *Slc25a34* KO primary myotubes under a fed condition (*P*-value < 0.05), related to Figure S3.

Metabolite	FC	$Log_2(FC)$	*P*-value	$-log_{10}(FDR)$
Proline	1.4468	0.532826	0.000271	1.529531
Adipate	2.4259	1.278525	0.000393	1.669156
Ethylmalonate	2.8738	1.522963	0.001932	1.153611
3-Methyl-2-oxovalerate	0.7294	-0.45527	0.002652	1.141066
Glycerol 3-phosphate	1.2776	0.35338	0.002726	1.226046
Ketoleucine	0.7259	-0.46223	0.003013	1.261722
Malonate	0.7011	-0.51235	0.003089	1.317889
N-Formylglycine	1.7859	0.836634	0.005306	1.140892
Serine	0.7592	-0.39735	0.009447	0.94151
trans-Aconitate	0.7872	-0.34519	0.01189	0.887388
Guanosine	1.5342	0.617502	0.014427	0.844777
Choline	1.5001	0.58506	0.020593	0.728029
Oxalate	0.7970	-0.32739	0.020917	0.75602
Fad	0.7513	-0.41253	0.022638	0.753862
Glutamine	0.8357	-0.25901	0.026266	0.71927
Mannose	0.8255	-0.2766	0.030791	0.678268
Threonine	0.8386	-0.25396	0.03082	0.704187
Aspartate	0.7507	-0.4137	0.032183	0.710225
Adenosine	1.3494	0.432318	0.033546	0.715683
Adenosine 3',5'-diphosphate	0.6440	-0.63497	0.033961	0.73262
Inosine	1.6030	0.680763	0.038461	0.699773
Nicotinamide	1.2965	0.374631	0.043227	0.669243
Glutamate	0.8461	-0.2411	0.045185	0.669304
3-Hydroxymethylglutarate	0.8464	-0.24059	0.049068	0.651986
Adenine	0.7791	-0.36006	0.049797	0.663311

Table S3

Differential mitochondrial metabolites in control and *Slc25a34* KO primary myotubes under a starved condition (P-value < 0.05), related to Figure 3.

Metabolite	FC	$Log_2(FC)$	P-value	$-log_{10}(FDR)$
Aspartate	0.5472	-0.8699	0.0000125	2.869176
Carnosine	0.5326	-0.909	0.0000366	2.704576
Choline	2.4536	1.294875	0.0000504	2.741555
Gluconate	0.5260	-0.92685	0.0000667	2.744247
Ethylmalonate	0.5229	-0.93528	0.0000671	2.838789
N-Formyl-L-methionine	0.6532	-0.6144	0.0000931	2.775676
Allose	0.6194	-0.69101	0.0001280	2.704559
Malate	0.6017	-0.73299	0.0001485	2.697935
Fumarate	0.5673	-0.81776	0.0002476	2.527041
Oxoglutarate	0.8159	-0.29359	0.0002750	2.527216
Threonine	1.4400	0.52608	0.0004816	2.325289
Glutamine	1.4903	0.575566	0.0006452	2.236086
3-Hydroxymethylglutarate	0.7922	-0.33603	0.0007638	2.197568
Phosphoenolpyruvate	0.6221	-0.6848	0.0009701	2.125873
Sorbose	0.6989	-0.51681	0.0010546	2.119566
Malonate	0.8115	-0.30127	0.0013285	2.047345
Phosphonoacetate	0.5684	-0.81497	0.0013468	2.067732
N-Acetylglycine	0.7415	-0.43146	0.0020475	1.910635
Mannose	0.7068	-0.50053	0.0022714	1.889033
Itaconate	0.8025	-0.31739	0.0023637	1.894019
Cytidine	1.6926	0.75926	0.0025780	1.877516
Pantothenate	0.5005	-0.99862	0.0026061	1.893014
Arginine	1.3342	0.415959	0.0031396	1.831431
D-Sedoheptulose	0.7030	-0.50848	0.0045538	1.688414
Guanosine	1.5948	0.67338	0.0052350	1.645598
trans-Aconitate	0.5308	-0.91367	0.0053389	1.6541
Lysine	1.7681	0.82224	0.0056608	1.645063
Inosine	1.3007	0.379285	0.0070240	1.567149
S-Adenosylhomocysteine	1.8604	0.895615	0.0072322	1.569701
Cytosine	1.7261	0.787539	0.0108769	1.407194
3-Hydroxybutanoate	1.2344	0.303784	0.0146194	1.29301
2-Hydroxybutyrate	1.4115	0.497222	0.0146343	1.306353
Adenine	0.7539	-0.40757	0.0166326	1.264131
Phosphocreatine	1.4125	0.498247	0.0184621	1.231775
Uridine monophosphate	0.6343	-0.65677	0.0197700	1.214638
Glycerol 3-phosphate	0.8654	-0.20856	0.0221267	1.177963
Isoleucine	1.5097	0.594294	0.0223815	1.184888
Histidine	1.2617	0.335312	0.0225612	1.192997
Citrate	0.7053	-0.50379	0.0231030	1.193973
trans-1,2-Cyclohexanediol	1.2701	0.344939	0.0238677	1.190826
Succinate	0.6632	-0.59256	0.0261289	1.162238

Uridine	1.4222	0.508174	0.0262875	1.170075
Methionine	2.7748	1.472378	0.0287390	1.141574
Adipate	0.8416	-0.24872	0.0316588	1.109534

Table S4

Differential whole-cell metabolites in control and *Slc25a34* KO primary myotubes under a starved condition (*P*-value < 0.05), related to Figure S3.

Metabolite	FC	$Log_2(FC)$	*P*-value	$-log_{10}(FDR)$
Sorbose	0.7251	-0.46377	3.19817E-05	2.457673
Inosine	1.4421	0.528212	3.83719E-05	2.67959
gamma-Aminobutyrate	0.7356	-0.44298	0.000157432	2.242603
Methylthioadenosine	0.7681	-0.38065	0.000172795	2.327103
Cytidine monophosphate	0.8175	-0.29077	0.000244872	2.272604
Adenine	0.7792	-0.35997	0.000344334	2.203745
Sorbitol	0.6689	-0.58003	0.000405724	2.199441
Oxalate	0.8039	-0.31495	0.000411093	2.251723
Adipate	0.4482	-1.15788	0.000417711	2.29594
Malonate	0.8139	-0.29704	0.000568926	2.207518
Uridine diphosphate-N-acetylglucosamine	0.7142	-0.48565	0.000839704	2.07984
Histidine	1.4653	0.551209	0.001348359	1.911949
Betaine	0.7053	-0.50369	0.001761089	1.830736
Sucrose	0.7713	-0.3747	0.00199262	1.809277
beta-Nicotinamide Adenine Dinucleotide	0.7455	-0.42374	0.002399434	1.758556
4-Guanidinobutanoate	0.8060	-0.31119	0.002873019	1.708355
Phosphocreatine	0.7618	-0.39243	0.00364187	1.631698
N,N,N-Trimethyllysine	0.8148	-0.29555	0.004890016	1.528536
Deoxycarnitine	0.7641	-0.38808	0.006405057	1.434804
Oxoproline	1.3700	0.454197	0.006428375	1.455502
Pyroglutamate	1.3700	0.454197	0.006428375	1.476692
CDP-ethanolamine	0.7998	-0.32232	0.006431727	1.496669
Proline	1.4298	0.515806	0.007031136	1.477276
Taurine	0.8033	-0.31606	0.011400605	1.285857
Choline	1.5207	0.604738	0.011741597	1.290786
Methionine	1.1438	0.193868	0.013801152	1.237632
Glutathione oxidized	1.2156	0.28162	0.015839088	1.194207
Phenylalanine	1.2943	0.372201	0.021233736	1.082705
L-Carnitine	0.8329	-0.26371	0.021998666	1.082575
Adenosine-monophosphate	0.7202	-0.47344	0.029065758	0.976313
3-Hydroxymethylglutarate	0.8475	-0.23871	0.032038752	0.94826
Guanosine	1.1805	0.239428	0.035450839	0.918097
Nicotinamide	0.8845	-0.17706	0.038795485	0.892306
Glutamine	1.1807	0.239645	0.049597741	0.798591

Table S5

Intracellular amino acid levels in control and *Slc25a34* KO primary myotubes under a fed condition, related to Figure 6.

Amino acid	FC	*P*-value
Proline	1.446761	0.000271
Serine	0.75925	0.009447
Glutamine	0.835661	0.026266
Threonine	0.83859	0.03082
Aspartate	0.750694	0.032183
Glutamate	0.846101	0.045185
Phenylalanine	0.867593	0.088441
Alanine	1.180707	0.358292
Histidine	1.087368	0.494701
Arginine	0.960353	0.656963
Leucine	1.030236	0.720458
Isoleucine	1.025551	0.725174
Lysine	0.973166	0.800691
Valine	1.142817	0.823766
Asparagine	1.009757	0.893008
Tryptophan	1.007604	0.92919
Methionine	1.00096	0.985921

Table S6

Intracellular amino acid levels in control and *Slc25a34* KO primary myotubes under a starved condition, related to Figure 6.

Amino acid	FC	*P*-value
Histidine	1.4653	0.001348
Proline	1.4297	0.007031
Methionine	1.1438	0.013801
Phenylalanine	1.2943	0.021234
Glutamine	1.1807	0.049598
Lysine	1.1699	0.081041
Threonine	1.1914	0.083601
Valine	1.3602	0.102727
Serine	1.1251	0.143351
Alanine	0.8758	0.310715
Leucine	1.0543	0.337289
Asparagine	1.0918	0.379808
Aspartate	1.0759	0.461471
Isoleucine	1.0492	0.47854
Arginine	1.0482	0.478971
Tryptophan	1.0321	0.646365
Glutamate	0.9547	0.684545

Table S7

Intracellular amino acid levels in control and *Slc25a35* KO primary myotubes under a fed condition, related to Figure 6.

Amino acid	FC	*P*-value
Threonine	1.3529	0.01786137
Glutamate	1.0978	0.09411465
Valine	0.8798	0.09809924
Methionine	0.8309	0.13839674
Aspartate	1.0577	0.18632468
Arginine	0.8879	0.23051777
Serine	1.0533	0.27774215
Glycine	1.0634	0.36862782
Glutamine	1.0804	0.37546956
Asparagine	1.0489	0.55186885
Alanine	1.0192	0.61704747
Leucine	1.0640	0.66619551
Histidine	0.9805	0.67797706
Tryptophan	1.0451	0.7548336
Phenylalanine	0.9617	0.76975901
Lysine	1.0247	0.78947997
Isoleucine	1.0237	0.81831771
Tyrosine	1.0106	0.83731928

Table S8

Intracellular amino acid levels in control and *Slc25a35* KO primary myotubes under a starved condition, related to Figure 6.

Amino acid	FC	*P*-value
Glutamate	1.2337	0.02481925
Phenylalanine	0.7522	0.04059091
Aspartate	1.1090	0.10800255
Arginine	0.6506	0.17986799
Threonine	0.9215	0.33515299
Glutamine	1.0274	0.4784421
Valine	1.4269	0.48237451
Leucine	1.0665	0.54554677
Isoleucine	1.0586	0.64544186
Alanine	0.9811	0.65888572
Serine	0.9524	0.66326534
Methionine	1.1417	0.81411647
Asparagine	1.0213	0.81919316
Histidine	1.0573	0.82182132
Tryptophan	0.9585	0.89250769
Tyrosine	0.9946	0.96015322

Table S9

gRNAs, ssDNA template and genotyping primers for generation of *Slc25a34*$^{flox/-}$ mice

**ssDNA template (loxP sequence)**
TTGGCCACGCTTACAGCCAAGACACCACAAGCCTAGGGCTTTTAGCTGGAGCCCTACAG<br>GGACAGGAGATGGAACAGGAAGTCTAGAGGACAAGGAGGGGTGCCCTCCCCTACTCTA<br>**ATAACTTCGTATAGCATACATTATACGAAGTTAT**GCTTGGCTCTTTCTTCCTGCCCACTG<br>TCCTTCCAGTCACAAGTGACCTTTCCTCCAGATCCCATGGCCCAGGCCCGCCCTGGCCA<br>GCAGCAGCTGGGATTGGTCTGAGCCAGAGGAGGTGTGTCCAGATTCCCATAGCCCTGC<br>TGATGAACAGCAGGTGGAGTTCCAAGACAACCAGGGGCCACCAGCCAGTGGCAGGATC<br>TCTGCCCTCTAGACATGGTGCTGGCCCCCTTTGATCCCCAGCCCTGTGACCATGCAGAG<br>GTACAGACTGCCTAGAGACTACCATCTATAGCCTGTGCCATGAAGCCAACTCAGGCACA<br>GATGGCTCCTGCCATGGATTCGAGGGAGATGGTGTCCCCGGCTGTGGACCTGGTGCTG<br>GGTGCCTCAGCCTGCTGCCTGGCCTGTGTATTCACCAACCCCCTGGAAGTGGTAAAGAC<br>CCGTCTACAACTGCAGGGGGAACTGCAGGCCCCAGGCACCTACCCACGGCCCTACCGG<br>GGCTTTGTGTCTTCTGTTGCAGCCGTGGCCCGGGCAGATGGGCTATGGGGCCTGCAGA<br>AGGGGCTGGCTGCTGGCCTTCTCTACCAGGGCCTCATGAATGGTGTCCGTTTCTACTGC<br>TATAGCCTGGCATGCCAGGCTGGCCTCACCCAACAACCAGGCGGCACTGTGGTCGCAG<br>GCGCTGCGGCTGGGGCATTGGGGGCCTTCGTGGGGAGTCCTGCTTACCTGGTAAGTGT<br>CTTCTCCTTCCTTTATAGCTCCGTGGCCAACGTGACCAGTGGACTTTGTGGCCCTGGGTT<br>GGGCGTGTCAGTTTGGGAGGGGACTGTTCGCTGGGAATATCTGGCTCAGACCTGAAATC<br>TGCCCTCACTCTGACCCTTGCTTCTTTGGGAGCCATGTCCACTGGTCTTTGCTGGGAGA<br>GGGAGCAGGTTATGGAGTAGGTAGATTCTAGTCCTCGCTTGC**ATAACTTCGTATAGCAT**<br>**ACATTATACGAAGTTAT**CTGTGGTGGCACCTAGGGAGCCTGGCATACAGTAAGAATAAA<br>ACCTGCCACAGAGCAGAGGGTTGTTGTGTCCTGGTCTAGCCACTGAATGCTTTACTCTGT<br>C

**gRNA sequences**	
gRNA-1	TGCCCTCCCCTACTCTAGCT
gRNA-2	TTCTAGTCCTCGCTTGCCTG

**Genotyping primers**		
	Forward primer	Reverse primer
5´ loxP	CGTCTATGGCCACCTGATCT	AGGGCTATGGGAATCTGGAC
3´ loxP	CTGCTGGCCTTCTCTACCAG	TACTGGGCTTATGGGTAGGC
HSA-Cre	ATGGTTGGGGAGGCCTTTGG	GGAAGCGAGGCTTCACTTGG

Table S10

RT-qPCR primer sequences

**Gene**	Forward primer	Reverse primer
Slc25a34	ATGGTGTCCGTTTCTACTGCT	AGCTGTGTCTTGACCAGGTA
Slc25a35	CAGGCAGCCTCTGAAATTG	TGGGTAGAGGAGCCGATG
Myod1	CCACTCCGGGACATAGACTTG	AAAAGCGCAGGTCTGGTGAG
Myogenin	GAGACATCCCCCTATTTCTACCA	GCTCAGTCCGCTCATAGCC
Myh4	TCTGGTAACACAAGAGGTGC	CATCTCAGCGTCGGAACTCA
Ppargc1a	AGCCGTGACCACTGACAACGAG	GCTGCATGGTTCTGAGTGCTAAG
Cox8b	GAACCATGAAGCCAACGACT	GCGAAGTTCACAGTGGTTCC
Gapdh	AAATGGTGAAGGTCGGTGTG	TGAAGGGGTCGTTGATGG
Rn18s	ACCGCAGCTAGGAATAATGGA	GCCTCAGTTCCGAAAACC
